# Oral contraceptive and breast cancer: do benefits outweigh the risks? A case – control study from Jordan

**DOI:** 10.1186/s12905-019-0770-x

**Published:** 2019-06-03

**Authors:** Sanaa K. Bardaweel, Amal A. Akour, Suha Al-Muhaissen, Husam A. AlSalamat, Khawlah Ammar

**Affiliations:** 1 Department of Pharmaceutical Sciences, School of Pharmacy, The University of Jordan, Amman, 11942 Jordan; 2 Department of Biopharmaceutics and Clinical Pharmacy, School of Pharmacy, The University of Jordan, Amman, 11942 Jordan; 3 Department of Pharmaceutics and Pharmaceutical Technology, School of Pharmacy, University of Jordan, Amman, 11942 Jordan; 4 King Hussein Cancer Center, Survey Unit, Amman, Jordan

**Keywords:** Oral contraceptives, Breast cancer, Risk factors, Contraception

## Abstract

**Background:**

Oral contraceptives (OCs) use has been linked to increased risk of breast cancer (BC) in several reports from the world. Limited number of similar studies have been conducted in the Middle Eastern female population. This study aimed to explore any possible correlation between the contemporary and duration of OCs use among Jordanian women and the risk of breast cancer.

**Methods:**

A case control study was conducted in 450 Jordanian women (225 as cases and 225 as controls), aged 18 to 65. Chi-square test was used to study the association between risk of breast cancer and different factors. Mann Whitney-U test was employed to evaluate the relation between time-dependent risk factor and breast cancer.

**Results:**

Our results indicated that regular use of OCs exhibited association with increased risk of breast cancer (OR = 2.25, 95% CI 1.34–2.79; *p* = 0.002), while the duration of OCs use was not associated with the increased risk of breast cancer (*p* > 0.05). In addition, other factors demonstrated significant association with the increased risk of breast cancer such as age at puberty, age at menopause, previous pregnancies, menopausal status, and family history of cancer.

**Conclusions:**

regular use of OCs may be associated with increased risk of breast cancer in Jordanian women. A larger sample size in multi-center setting study is required to confirm this finding among the Jordanian female population.

**Electronic supplementary material:**

The online version of this article (10.1186/s12905-019-0770-x) contains supplementary material, which is available to authorized users.

## Background

Breast cancer (BC) is the most prevalent type of cancer affecting females in both the developed and developing countries [1]. Worldwide, it has been reported that about 1.4 million new cases are diagnosed annually with nearly close to10% of the newly diagnosed cases are women under the age of 40 [2, 3]. In Jordan, cancer is the second most common cause of death, preceded by cardiovascular diseases, with breast cancer being the most frequently occurring type of cancer among Jordanian females [4]. Approximately, 15% of the newly diagnosed cases in Jordan are women under 40 years [5].

The significant increased concern of breast cancer, worldwide and in Jordan, may be attributed to the prolonged life expectancy and the adoption of the western lifestyle with its related risk factors [6]. Genetic mutations, alcohol consumption, obesity and physical inactivity were all related to increased breast cancer risk [7, 8].Interestingly, long-term utilization of oral contraceptives (OCs) has been associated with a small increase in breast cancer risk in young women [9]. A 2013 meta-analysis estimated the lifetime absolute risk increment of breast cancer linked with ever versus never use of OCs at about 0.89% [9]. The International Agency for Research on Cancer came to conclusion that there was enough evidence to support combined estrogen–progestin OCs carcinogenicity in humans, with an increased breast cancer risk limited to women who were currently utilizing or had recently utilized them [10]. Data argue whether this risk is increased in women with family history of breast cancer [11] or without influence of family history of breast cancer [12].

Globally, around 140 million women worldwide utilize oral contraceptives; this portion approximately accounts for 13% of women between 15 and 49 years old [13]. Among Jordanian women, intrauterine devices are the most preferred method of contraception followed by OCs [14]. Unfortunately, there is scarcity of data exploring hormonal factors as intensifying risk for breast cancer in the Middle Eastern populaces, particularly in Jordan. For this reason, we performed an expanded case control study to examine the probability of such interaction, if exists, in the Jordanian female population.

## Methods

### Study participants

Ethical approval to perform the study was obtained from the Scientific Committee at the King Hussein Cancer Center (IRB:17KHCC61). Participants’ information remained confidential and within the institution. The study sample was selected via a non-random sampling method. All women who have been diagnosed with breast cancer (cases, *n* = 225) and who visited King Hussein Cancer Center breast cancer clinics between April and November 2017 were approached. Control cases were females, free of breast cancer that were matched for age to the study participants and they were selected conveniently from other clinics like ambulatory care, family medicine and gynecology. Entitled participants (cases and controls, *n* = 450) were women in the age category of 18 to 65 years old Jordanian females. Only participants who were able to provide an informed consent, based on a standard written statement, were eligible to fill the questionnaire and become part of the study. Information about history of breast cancer or any type of cancer, age at first child, and duration of breastfeeding, abortion, and age at menopause, smoking, and physical activity were all collected. In addition, participants were asked to provide detailed information regarding the use of OCs or any other hormonal replacement therapy. Demographic data were also obtained from all participants.

### Data collection

The questionnaire was structured based on initial discussions with health care professionals at the KHCC breast cancer clinics and careful inspection of literature [Additional File 1]. A group of four clinical pharmacists performed the interviews at the KHCC with the participating women confirming privacy and confidentiality of participants due to the sensitivity of the study population. All pharmacists were trained and instructed by the principle investigator (PI) to certify delivering the questionnaire in similar manner.

### Statistical analysis

All data were coded, listed and analyzed using Statistical Package for Social Sciences program (SPSS®) database for Windows, version 23 (IBM, Chicago, IL). Kolmogorov Smirnov test was used to study the normal distribution of variables. The association between the incidence of physician diagnosed breast cancer and different factors was detected using Chi-square test. Mann Whitney-U test was used to assess the relation between time-dependent risk factor and breast cancer. Binary logistic regression was used to study the correlation between the incidence of breast cancer and possible risk factor, also to measure the degree of contribution of these risk factor to breast cancer. *P*-value < 0.05 was used to indicate statistical significance.

## Results

A total of 450 women, including women who have been diagnosed with breast cancer (cases, *n* = 225) and women who don’t have breast cancer (controls, *n* = 225), were interviewed. Characteristics of the study population, demographics and descriptive properties of the sample, as education, the area of residence, occupation, income, marital status, and number of children, if any, are listed in Table 1. Comparison data were represented by numbers and percentages. The vast majority of both cases and control samples were high school or higher educated (> 70%), housewives (50% or more), and married (65% or more).

**Table 1:** Demographic and Characteristics of the Study Population (*N* = 450)

	Cases (*n* = 225) *N* (%)	Controls (*n* = 225) *N* (%)
Education (*N* = 449)		
Uneducated	2 (0.89	22 (9.44)
Elementary	22 (9.82)	42 (18.03)
High School	42 (18.75)	61 (26.18)
College	75 (33.48)	56 (24.03)
BSc^1^	73 (32.59)	44 (18.88)
Postgraduate	10 (4.46)	8 (3.43)
Residence (*N* = 448)		
Amman	162 (72.65)	75 (33.33)
Other provinces	61 (27.35)	150 (66.67)
Employment (*N* = 449)		
Employee/Job owner	72 (32.14)	78 (34.67)
House Wife	133 (59.38)	112 (49.78)
Retired	19 (8.48)	15 (6.67)
Unemployed	0 (0)	20 (8.89)
Monthly Income in JDs (*N* = 442)		
< 500	77 (35.48)	117 (52)
500–1000	114 (52.53)	99 (44)
> 1000	26 (11.98)	9 (4)
Number of Dependents (*N* = 448)		
≤ 5	155 (69.51)	140 (62.22)
> 5	68 (30.49)	85 (37.78)
Marital Status (*N* = 449)		
Single	42 (18.7)	36 (16)
Married	153 (68.3)	173 (76.89)
Divorced	13 (5.8)	4 (1.78)
Widowed	16 (7.14)	12 (5.33)
Parity (*N* = 450)		
Yes	163 (72.44)	173 (76.89)
No	62 (27.56)	52 (23.11)
Number of Children (*N* = 450)		
No	56 (24.89)	52 (23.11)
< 4	80 (35.56)	53 (23.56)
4–8	83 (36.89)	113 (50.22)
> 8	6 (2.67)	7 (3.11)
Chronic Diseases^a^ (*N* = 445)		
Hypertension	25 (11.85)	44 (22.11)
Diabetes Mellitus	3 (1.42)	9 (4.52)
Osteoporosis	9 (4.27)	1 (0.5)
Morbid Obesity	5 (2.37)	0 (0)
Blood Diseases	1 (0.47)	0 (0)
Others	6 (2.84)	14 (7.04)
None	162 (76.78)	131 (65.83)
Thyroxin Therapy (*N* = 447)		
Yes	24 (10.81)	12 (5.330
No	198 (89.19)	213 (94.67)
Tamoxifen Therapy (*N* = 446)		
Yes	80 (36.04)	0 (0)
No	142 (63.96)	224 (100)
Reasons of Oral Contraceptives Use (*N* = 446)		
Pregnancy Control	62 (23.57)	37 (16.59)
Stop Period	3 (1.14)	8 (3.59)
Others	23 (8.75)	3 (1.35)
Nonuser	175 (66.54)	175 (78.48)
Oral Contraceptives Prescriber (*N* = 136)		
Physician	67 (76.14)	44 (99.67)
Nurse	0 (0)	1 (2.08)
Pharmacist	14 (15.91)	3 (6.25)
Others	7 (7.95)	0 (0)
Getting MD^1^ Counseling (*N* = 27)		
Yes	3 (13.04)	0 (0)
No	20 (86.96)	4 (100)

^a^ more than one disease can be selected

^1^*BSc* Bachelor of Science

^1^*MD* Medical Doctor

As demonstrated in Table 2 and Table 3, a comparison between cases and controls in term of risk factors revealed that no significant differences were detected between cases and controls with regard to age groups, breastfeeding and breastfeeding duration, history of miscarriage, regularity of period, previous use of hormonal therapy for menstrual cycle irregularities, the type of contraceptive pills, whether it was combined hormonal or only progesterone type, and age when the first child was born.

**Table 2:** Risk Factors in the Study Population, *p*-values from Chi-square test for Comparison between Cases and Controls

Factor	Cases (*n* = 224) *N* (%)	Controls (n = 225) *N* (%)	*p*-value^1^
Age in years (*N* = 449)			
18–35	31 (13.84)	31 (13.78)	0.988
36–45	89 (39.73)	91 (40.44)	
46–65	104 (46.43)	103 (45.78)	
Breastfeeding (*N* = 450)			
Yes	144 (64)	154 (68.44)	0.522
No	25 (11.11)	19 (8.44)	
N/A^*^	56 (24.89)	52 (23.11)	
Duration of breastfeeding in years (*N* = 449)			
≤ 1	50 (22.32)	61 (27.11)	0.610
> 1	94 (41.96)	93 (41.33)	
No	24 (10.71)	19 (8.44)	
N/A^*^	56 (25)	52 (23.11)	
Pregnancy (*N* = 450)			
Yes	115 (51.11)	173 (76.89)	0.000
No	110 (48.890	52 (23.11)	
Miscarriage (*N* = 450)			
Yes	92 (40.89)	84 (37.33)	0.249
No	133 (59.11)	141 (62.67)	
Menopausal Status (*N* = 450)			
Premenopausal	101 (44.89)	74 (32.89)	0.006
Postmenopausal	124 (55.11)	151 (67.11)	
Regularity of Period (*N* = 449)			
Yes	190 (84.82)	203 (90.22)	0.056
No	34 (15.18)	22 (9.78)	
Personal history of cancer (ovarian, rectal, etc.) (N = 449)			
Yes	27 (12.05)	0 (0)	0.000
No	197 (87.95)	225 (100)	
First or second – degree relative have cancer (*N* = 449)			
Yes	109 (48.66)	34 (15.11)	0.000
No	115 (51.34)	191 (84.89)	
Smoking Status (*N* = 448)			
Past Smoker	25 (11.21)	1 (0.44)	0.000
Current Smoker	11 (4.93)	25 (11.11)	
Never Smoked	187 (83.86)	199 (88.44)	
Hormonal Therapy for Menstrual Irregularities (*N* = 447)			
Yes	24 (10.81)	13 (5.78)	0.060
No	198 (89.19)	212 (94.22)	
Postmenopausal Hormonal Therapy (i = 448)			
Yes	8 (3.59)	0 (0)	0.004
No	215 (96.41)	225 (100)	
Regular Use of Oral Contraceptives (*N* = 446)			
Yes	87 (39.01)	48 (21.52)	0.000
No	136 (60.99)	175 (78.48)	
Oral Contraceptives Type (*N* = 81)			
COC^*^	31 (49.21)	10 (55.56)	0.063
POC^*^	32 (50.79)	8 (44.44)	

^*^N/A Not Applicable

^*^POC Progestin-Only Contraceptives

^*^COCs Combined Oral Contraceptives

^1^Chi-square test

**Table 3:** Study Sample Characteristics in Two Groups, with Differences in Medians Expressed as Nonparametric t-test *p-values*

Parameter	Group	N	Median (IQR)	*p*-value^c^
Age at 1st child	Cases	169	22(20–26)	0.765
	Control	173	23(20–25)	
Number of Miscarriage Incidences	Cases	122	1(0.75–2.0)	0.004
	Control	85	1.3(1–2)	
Age at Puberty	Cases	222	13(12–14)	0.000
	Control	225	14(13–15)	
Age at Menopause	Cases	101	48(40.5–51)	0.000
	Control	74	50(49–50)	
Duration of Oral Contraceptives Use^a^	Cases	211	6.6 (4.2–9.1)^b^	0.002
	Control	223	4.1 (2.4–5.8) ^b^	

^a^Duration recorded for the longest continuous use

^b^Median (IQR)

^c^Mann-Whitney Test

Nonetheless, significant differences were observed between cases and controls in terms of previous pregnancy (*p < 0.001)*, menopausal status (*p < 0.01)*, personal history of cancers other than breast cancer (*p < 0.001)*, family history of cancer; first or second degree relatives (*p < 0.001)*, smoking status (*p < 0.001)*, use of hormonal replacement therapy (*p < 0.01)*, number of previous miscarriages (*p < 0.01)*, age at puberty and menopause (*p < 0.001)*, and the use of OCs (*p < 0.001)* as well as the duration of their use (*p < 0.01)* (Table 2 and Table 3). Additionally, OC users represent 39% of cases and 21.5% of controls (including both regular and intermittent use), while median use duration in months was 6.6 months for cases and 4.1 months for control.

Factors that have baseline significant difference between cases and controls were inserted into logistic regression utilizing stepwise method to test for significant association with the incidence of breast cancer (Table 4). After adjustment for possible confounders, regular use of OCs showed association with incidence of breast cancer (OR_adjusted_ = 2.25, 95% CI 1.34–2.79; *p* = 0.002), while the duration of OCs use did show no association with the incidence of breast cancer (*p* > 0.05). Other factors that demonstrated significant association with the incidence of breast cancer are listed in Table 4. Interestingly, age at puberty, age at menopause, previous pregnancies, menopausal status, and family history of cancer were all among the factors that had significant relationship to breast cancer incidence.

**Table 4:** Tested Model Results Using Stepwise Logistic Regression Analysis

Factor^a^	*p-value*	OR (CI)
Regular Use of Oral Contraceptive^b^	0.000	2.33 (1.54–3.55)
Regular Use of Oral Contraceptive^c^	0.002	2.25 (1.34–2.79)
Age at Puberty	0.005	0.23 (0.08–0.64)
Age at Menopause	0.039	0.64 (0.42–0.98)
Pregnancy (Yes-No)	0.000	0.21 (0.12–0.34)
Menopausal Status (Premenopausal - Postmenopausal)	0.004	2.11 (1.27–3.49)
First or second – degree relative have cancer	0.000	4.07(2.42–6.83)

^a^Only factors of significant effect in the tested model were shown

^b^Unadjusted

^c^Adjusted

## Discussion

To our knowledge, this is the first case-control study to evaluate the link between the type and duration of contemporary oral contraceptives use and breast cancer risk in Jordanian women. The need for safe and effective contraception is a concern shared by women worldwide, including Jordanian women. While, OCs were viewed as convenient form of contraception [14], there were concerns about their safety; 40.8% of Jordanian women thought that OCs are not safe, and 75.1% had experienced various side effects [14].

A total of 5013 Jordanians and 2441 non-Jordanians residing in the Kingdom were diagnosed with cancer in 2012, according to the Health Ministry’s annual statistics report for 2015 [15] with about 2667 Jordanian women among those diagnosed, constituting 53.2%. Breast cancer is the most common form of cancer among Jordanian females, with 994 diagnoses in 2012 (37.3%), while lung cancer was the most common type of cancer among Jordanian males, with 282 cases diagnosed in the same year (12.4%) [15]. There were few attempts to understand the risk factors of breast cancer in Jordanian women. A very recent hospital-based multi-center case-control study by Al-Qadiri et al (2018) [16] has evaluated the risk factors of breast cancer in Jordanian women. Cases were older and had higher education than controls. Good lifestyle habits, such as exercising and eating healthy diet that focuses on fruits and vegetables, were associated with lower risk of breast cancer. Similar to our findings, Al-Qadiri et al found out that history of breast cancer in the first relatives is significantly associated with higher risk of breast cancer, but smoking, was not associated with breast cancer risk. However, the relationship between the use of OC use and BC risk was not assessed in Al-Qadir study. In a relatively less recent study in Jordan [17], it was found that the use of fertility drugs, irregular periods, exposure to pesticides, chemicals and breast trauma were among the significant risk factors. Consistent with our findings, the earlier age of menarche and menopause and the use of hormonal therapy were associated with higher BC risk. Interestingly, the use of OCs was significantly associated with the risk of breast cancer (OR = 6.28, 95% CI: 3.14–12.55) [17]. However, the link between the type and duration of contemporary OC use and BC risk in Jordanian women has not been investigated yet.

According to the results from the current study, women who used OCs regularly were at higher risk of developing breast cancer than their nonuser counterparts (OR_Adjusted_ = 2.25; 95% CI 1.34–2.79; *p* = 0.002), with no significant difference according to the type of OCs used, whether it was a combined oral contraceptive (COC) or progestin-only contraceptive (POC). Surprisingly, the risk was not increased with the duration of OCs use. Other risk factors include age at puberty, age at menopause, previous pregnancies, menopausal status, and family history of cancer.

Results from our study is consistent with previous ones. In 1996, a combined analysis encompassing about 90% of studies all over the world with 53,297 BC cases and 100,239 women without BC was published by the Collaborative Group on Hormonal Factors in Breast Cancer. It showed that there was a moderate increase in the relative risk of breast cancer of current COC users versus those who never used them. However, this risk diminishes proportionally with the number of years after last use, and then it disappears totally after 10 years of stopping the use [12]. This relative risk was only slightly affected by identified risk factors of breast cancer such as age of menarche, menopause status and alcohol use. Comparably in our study, although these factors were significantly associated with OR of breast cancer, they had a small effect on OR of BC risk (2.33 vs. 2.25 of unadjusted vs. adjusted OR, respectively). Comparable to our finding, a statistically significant risk difference by oral contraceptive type or formulation was not found. Nevertheless, since the majority of the cases in the former study were diagnosed in the 1980s, it is expected that they use OC formulations that are different from the contemporary ones, even more, half of the studies were missing formulations data. A recent study, with smaller sample size “the Nurses’ Health Study II”, reported an increased in the risk of BC (RR = 1.3; 95% CI, 1.0–1.7) associated with current OCs use in general, especially with the use of one triphasic OCs (OR = 3.1; 95% CI, 2.0–4.7) [18], after controlling for potential or know risk factors in their multivariate analysis, including but not limited to, age at menarche, family history of the cancer, menopausal status and current pregnancies, which were found to be significantly associated with the risk of BC in our study. Whereas the Women’s Contraceptive and Reproductive Experiences (CARE) Study, found the BC risk was not associated with current use of OCs [19] even when considering different types of OCs. But the (CARE) Study, in agreement with ours, found a significant different in cases and controls in age at menarche, menopausal status, number of pregnancies, use of hormonal therapy and family history. Recently, and based on pharmacy dispensing traces of women enrolled in a huge U.S. health plan, a study by Beaber et al evaluated the modern use of contemporary OC formulations used from 1989 to 2009 in the US. They reported that recent OCs use (within the last year) was associated with an increased BC risk (OR = 1.5; 95% CI, 1.3–1.9) relative to never or former OCs use. Contrasting our study, however, the use of contemporary OCs types, i.e. with low-dose estrogen, was not significantly associated with BC (OR = 1.0; 95% CI, 0.6–1.7). Nevertheless, the Beaber study had relatively short durations of continuous enrollment before reference date, so the exposure length to OCs could not be evaluated. Accordingly, the “never and former” OC users comprised the reference group, while in our analysis, the former users of OCs were included in the exposure group. In contrary to our study, where we collected data on potentially relevant confounders for all women, data on the possible confounders were unavailable for all women in the former study. Also, in Beaber et al study there were several analyses by OC type, and it is probable that some of the statistically significant findings they had may be due to chance as a result of the number of analyses performed.

Recently a very large-scale observational study from Denmark examined the association between the low-dose, more contemporary formulations of hormonal contraception [20] including progestin-only contraceptives, and the risk for breast cancer in women younger than the age of 50. In concordance with our results, the study found that there is an increase in risk, yet small, (about a 20%), but in contrast to ours, the risk was proportional with duration of use. Also, this risk may persist after five years of cessation in women who used hormonal contraception for a minimum period of 5 years. The study by Mørch et al (2017) had much larger sample size than ours which allowed them to have sizable subgroups according to the type of OCs used. However, they did not adjust for potential factors with correlation to breast cancer such as age at menarche and breast-feeding or smoking status, which would have affected the calculated risk. Also, they lacked information about the exposure to OCs before study entry, but we have this type of information because the case-control type of study.

Limitations of our study include the retrospective type of research design which might have created a recall bias in some of the variables; for example, age of menarche, age of menopause and duration of OCs use. In addition, not all women in this study could self-report the type of OCs they have used and thus analysis based on the different combination doses was not possible. Those factors, along with the limited sample size and single-centered nature of study, make results of the current study ungeneralizable. However, the study focuses on the relationship between type and duration of OCs use and other risk factors with breast cancer in Jordanian women.

## Conclusions

This study is the first report in Jordan to investigate the correlation between the contemporary and duration of use of OCs among Jordanian women and the risk of breast cancer. In addition to regular OCs use, significant differences in correlation of breast cancer were found between the case and the control groups, especially for the following risk factors: age at puberty, age at menopause, previous pregnancies, menopausal status, and family history of cancer. Future studies should include a larger sample size in multi-center setting all over the Kingdom. Also, the relationship between OCs use and specific subtypes of breast cancer [Estrogen Receptor-Positive (ER^+)^, Estrogen Receptor-Negative (ER^-)^, Human Epidermal growth factor Receptor 2-Positive (HER2^+^), Progesterone Receptor-Positive (PR^+^)] should be evaluated.

Findings from this study have implications in clinical practice. It is important for women to have this information before they decide to start using OCs. Healthcare providers should assist women in decision-making about alternative contraception methods such as intrauterine devices without hormone release, tubal ligation, and vasectomy for their partner. This decision needs to be made only after a careful discussion of benefits of contraception versus risks.

## Additional file

Additional file 1: Questionnaire (PDF 204 kb)

## Data Availability

The data that support the findings of this study are available from the corresponding author on reasonable request.

## Abbreviations

BC: Breast Cancer
CARE: Contraceptive and Reproductive Experiences
COC: Combined Oral Contraceptive
ER^−^: Estrogen Receptor-Negative
ER^+^: Estrogen Receptor-Positive
HER2^+^: Human Epidermal growth factor Receptor 2-Positive
KHCC: King Hussein Cancer Center
OCs: Oral Contraceptives
PI: Principle Investigator
POC: Progestin-Only Contraceptive
PR^+^: Progesterone Receptor-Positive
