# Supplementary material for: Oral contraceptive and breast cancer: do benefits outweigh the risks? A case – control study from Jordan
Source: BMC Womens Health. 2019 Jun 3;19:72. doi: 10.1186/s12905-019-0770-x (PMC6547482; doi:10.1186/s12905-019-0770-x)
Supplement: Supplementary file 1 — Questionnaire (PDF 204 kb) [file 12905_2019_770_MOESM1_ESM.pdf]

Research study: Oral contraceptive and breast cancer: do benefits outweigh the risks? a Case – control study from Jordan

Dear Ladies,

We thank you for your trust and visit the King Hussein Cancer Center wishing you a speedy recovery. Please be advised that we are conducting a survey on women who have been diagnosed with breast cancer at the KHCC to investigate a link between breast cancer and the use of birth control pills. The main researcher of this study is Dr. Sanaa Bardaweel, School of Pharmacy, University of Jordan**.**

Any information collected through surveys will be used in reports only on an aggregate basis. That is, it will not be possible for anyone to identify a particular individual with any set of responses. We strive to ensure that data is kept secure, and that we collect only as much personal data as is required for the survey. We assure you that your responses will be kept confidential. The non-participation in this research will not affect the service provided in the center.

Your participation is expected to last up to ten minutes, and your participation will not result in any inconvenience or adverse effects**.**

This form has been reviewed and approved by the Institutional Committee at King Hussein Cancer Center. The Institutional Committee is a committee authorized by the Food and Drug Administration to review, monitor medical research and clinical studies to maintain the safety of participants within the framework of the study and ensure the preservation of their rights.

If you have any general questions or questions concerning the rights of the participant, you should contact the Institutional Committee at telephone number 5300460 ext. 1669.

In the case of any inquiry about the study or specific questions related to this study and in case of any complications related to the study please contact: Dr. Sanaa Bardaweel at telephone number 0795042395

Code: Date:

Personal information			
1	**Age**		
	☐ 18-25 years ☐ 26-30 years ☐ 31-35 years ☐ 36-40 years ☐ 41-45 years ☐ more than 46 years		
2	**Educational level**		
	☐ Uneducated ☐ Primary ☐ Secondary ☐ Diploma ☐ Graduate ☐ Postgraduate studies		
3	**Nationality**		
	☐ Jordanian	☐ Non-Jordanian, please mention	
4	**Place of residence / residence**		
	☐ Amman ☐ Alzarqa ☐ Irbid ☐ Other, please mention		
5	**Work status**	☐ I work ☐ Housewife	☐ Retired
6	**If you are a worker, please specify the nature of the work**		
7	**Monthly income of the family**		
	☐ Less than 500 dinars ☐ from 500-1000 dinars ☐ more than 1000 dinars		
8	**Number of family members benefiting from income**		
	☐ 1-2 ☐ 3-5 ☐ 6-10 ☐ More than 10		
9	**Social status**		
	☐ Single ☐ Married ☐ Divorced ☐ Widowed		
10	**If you are a married person, please specify the duration of the marriage**		
	☐ Less than one year ☐ years - 2 years ☐ 3-5 years ☐ more than 5 years		
11	**Have you ever had children?**	☐ Yes, skip to Question 12	☐ No, skip to Question 16
12	**Age at first child**		
13	**Number of children**		
14	**Have you breastfed your children?**	☐ Yes	☐ No
15	**Average duration of breastfeeding in months**		
	☐ Less than 6 months ☐ 6-12 months ☐ 12-18 months ☐ 18-24 months ☐ More than 24 months		
16	**Has there been a pregnancy before**	☐ Yes, skip to Question 17	☐ No, skip to Question 19
17	**Was there a previous miscarriage?**	☐ Yes	☐ No
18	**If yes, please specify the number of times**		
19	**Age at first menstrual cycle; Age at puberty**		
20	**Age when the cycle is interrupted**	**year/s**	**☐ It wasn't interrupted**
21	**Is your period regular?**	☐ Yes	☐ No
**Patient history**			

22	**Have you ever had any type of cancer?**	☐ Yes	☐ No
23	**If yes, please specify the type of cancer**		

24	**Have your first-degree relatives (father, mother, brothers, sisters, children) or second-degree (uncles, aunts, etc.) experienced any of the following cancers: colon, prostate, uterus, ovaries?**
	☐ Yes ☐ No

**Degree of kinship:**	**Type of cancer:**
25	26
27	28
29	30

31 **Do you suffer from any of the following chronic diseases?**

☐ Hypertension ☐ Diabetes ☐ Osteoporosis ☐ Obesity ☐ Hematology

☐ Other, please specify…

32 **Smoking "cigarettes only" does not include hookahs, are you…**

☐ Previously smoked ☐ Currently smoker ☐ I have never smoked

33 **If you are a smoker or have you smoked before, what is the number of smoked cigarettes per day?**

☐ Less than 1 pack / day ☐ 2 packs per day ☐ More than 2 packs per day

Hormonal Therapy; have you ever taken any of the following hormones for therapeutic reasons?

	**The hormone**			**When**	**Duration**
34	Hormones regulate the cycle	☐ Yes	☐ No		
35	Hormones after cycle interruption	☐ Yes	☐ No		
36	Treatment of hormonal therapy, Thyroxine	☐ Yes	☐ No		
37	Hormone therapy, Tamoxifen	☐ Yes	☐ No		

Pregnancy regulation

38 **Have oral contraceptives been used?**

☐ Yes, please go to Question 39 and follow the questionnaire

☐ No, please go to Question 47 and add any observations related to the subject matter if any, and return the questionnaire to the researcher.

39 **What are the reasons for using oral contraceptive pills?**

☐ to spacing pregnancy ☐ to prevent the menstrual cycle ☐ Other, mention them

40 **What is the longest period of continuous use of oral contraceptive pills?**

41 **If you have used the pills sporadically, please specify how long have you used them.**

☐ Less than six months ☐ Six months - One year ☐ One and a half years ☐ Two years

42 **Who prescribe you these pills?**

☐ Doctor ☐ Midwife / Nurse ☐ pharmaceutical / pharmaceutical

☐ Health education (home visits) ☐ Other Please specify

43 **If your doctor did not prescribe the contraceptive pills, have you consult your doctor before using the contraceptive pills?**

☐ Yes ☐ No

44 **Before prescribing pills, have you been asked about breast cancer incidence among your relatives?**

☐ Yes, skip to Question 45 ☐ No, skip to Question 46

45 **If you have relatives with breast cancer, or there is a family history, and your service provider was informed, have you been prescribed the pills?**

☐ Yes, prescribed it ☐ No, did not prescribe it

46 **Which type of pill did you use? you can choose more than one answer**

☐ Diane 35 ☐ Gracial

☐ Progyluton ☐ Cerazette

☐ Marvelon ☐ Zahra

☐ Climen ☐ Yasmin

☐ Yaz ☐ Primolut Nor

47 **Notes you'd like to share with the research team.**

Thank you for your cooperation

We sincerely wish you good health
